# The Impacts of Crystalline Structure and Different Surface Functional Groups on Drug Release and the Osseointegration Process of Nanostructured TiO_2_

**DOI:** 10.3390/molecules26061723

**Published:** 2021-03-19

**Authors:** Anna Pawlik, Magdalena Jarosz, Robert P. Socha, Grzegorz D. Sulka

**Affiliations:** 1 Faculty of Chemistry, Jagiellonian University, Gronostajowa 2, 30387 Krakow, Poland; pawlika@chemia.uj.edu.pl (A.P.); sulka@chemia.uj.edu.pl (G.D.S.); 2 Jerzy Haber Institute of Catalysis and Surface Chemistry, Polish Academy of Science, Niezapominajek 8, 30239 Krakow, Poland; ncsocha@cyf-kr.edu.pl

**Keywords:** titanium dioxide layers, surface modification, silane derivatives, heat treatment, cell response

## Abstract

In implantable materials, surface topography and chemistry are the most important in the effective osseointegration and interaction with drug molecules. Therefore, structural and surface modifications of nanostructured titanium dioxide (TiO_2_) layers are reported in the present work. In particular, the modification of annealed TiO_2_ samples with —OH groups and silane derivatives, confirmed by X-ray photoelectron spectroscopy, is shown. Moreover, the ibuprofen release process was studied regarding the desorption-desorption-diffusion (DDD) kinetic model. The results proved that the most significant impact on the release profile is annealing, and further surface modifications did not change its kinetics. Additionally, the cell adhesion and proliferation were examined based on the MTS test and immunofluorescent staining. The obtained data showed that the proposed changes in the surface chemistry enhance the samples’ hydrophilicity. Moreover, improvements in the adhesion and proliferation of the MG-63 cells were observed.

## 1. Introduction

Modern medicine greatly depends on medical implants applied to support or replace damaged organs or tissues. They are made of one or more biomaterials, which have to be biologically indifferent and non-toxic, and not cause inflammatory reactions or produce harmful substances, but must improve the interaction between the implant surface and surrounding tissues [1]. What is more, their shape and mechanical properties should be suitable for the implantation site [1]. Some of the most commonly used materials for endoprostheses are titanium (Ti) and its alloys because of their excellent mechanical and biological properties and corrosion resistance [1,2,3,4]. However, their implantation is associated with a long-term integration process with tissues, leading to side effects such as inflammation and even implant recoil [1,5]. Therefore, it is necessary to modify their surfaces to improve the processes of osseointegration [1,5,6]. A stable and porous layer that firmly adheres to the substrate, such as titanium dioxide obtained by the anodization process, is one such modification [1,5].

Anodic TiO_2_ (ATO) layers can be further modified to improve the biomaterial interaction with the cells and alter the drug release process. Amorphous TiO_2_ can be functionalized with sodium hydroxide (NaOH) [7,8,9,10,11], which results in better hydrophilicity of the surfaces, and thus, improvements in the adsorption of water and protein molecules, and consequently, the cell adhesion [7,12]. Another way of modifying the samples is coating them with self-assembled monolayers (SAMs), i.e., coatings resulting from the adsorption of organic molecules from a solution [13]. Due to hydroxyl groups (-OH) on the TiO_2_ surface, it is possible to form chemical bonds between them and organic monolayers [13]. Some such compounds are silane derivatives, which possess different functional groups, such as amino (-NH_2_), thiol (-SH), carboxyl (-COOH), and glycidyl (-CH-(O)-CH_2_) groups [13,14,15]. Their presence can considerably increase drug delivery control—e.g., take ibuprofen [16]—and improve interactions with cells, e.g., fibroblasts [17]. What is more, their adsorption on the TiO_2_ surface is almost irreversible [18] and alters the wettability of the samples [19].

To enhance the biomaterial’s biocompatibility, we can also modify its surface and crystalline structure. The as-prepared anodic TiO_2_ layers are amorphous [1,20,21]; however, simple annealing of the materials at the appropriate temperature leads to a phase transition to anatase, rutile, or mixed phases [1,20,21,22]. The thermal treatment of anodic nanostructures also leads to decreased fluorine content, which may play a role in the interaction between the biomaterial and cells [20,23]. Among all forms, anatase possesses the best antibacterial properties [24] and may impact drug release from ATO layers [25].

As shown in our previous articles [11,25,26], anodic nanoporous TiO_2_ layers with different crystalline structures can be used as potential drug delivery systems for compounds with varied structures and water solubility. Typically, the release profiles of, e.g., ibuprofen and gentamicin from amorphous ATO layers, are characterized by a burst release stage, followed by continuous delivery of the medicine [11,25,26]. There are many possibilities for suppressing the initial burst effect, among which changing the crystalline structure [25,26] and functionalization of the TiO_2_ surface [11,27,28,29] are the most commonly exploited.

Biomaterials have to provide rapid and robust connections with the surrounding tissues to avoid implant rejection [30]. The initial interaction between cells and a biomaterial surface plays a crucial role in cell adhesion, spreading, migration, proliferation, and differentiation [7,31,32,33,34]. Within the first seconds in the in vitro or in vivo conditions, the adsorption of water and proteins from the cellular medium or body fluids occurs [30,35]. This process is affected by surface properties, such as wettability, surface energy, crystalline structure, electrical properties, and the presence of nano- or microstructures or functional groups [30,33,36,37]. The advantage of nanostructured surfaces over the flat titanium sample is due to the increased surface roughness at the nanoscale level, which is combined with locally increased charge density at the surface irregularities, which promotes protein adsorption [36,37]. What is more, the protein adsorption is followed by the interaction between cells and implant surface using the van der Waals forces [4]. Later, bonds between the cell surface receptors (integrins) and extracellular matrix proteins are formed [4,12,30], which further leads to the regulations of the cell functions [12]. Then, the cell’s cytoskeleton is reorganized to form filopodia and finger-shaped projections, making it possible to move across the implant surface [4,30]. As mentioned above, the functionalization of the TiO_2_ surface can cause differences in cell behavior on the examined samples. For example, NaOH-modified layers exhibit increased hydrophilicity and surface roughness that should improve the biomaterial cell response, but the release of sodium ions may deteriorate cell adhesion and proliferation [7]. Additionally, the presence of silane derivative monolayers on TiO_2_ layers affects cell behavior [12,17]. For instance, 3T3 fibroblasts adhere better to the samples modified with (3-aminopropyl)trimethoxysilane than those modified with (3-mercaptopropyl)trimethoxysilane [17].

Although it was proven that the change in the crystalline structure of TiO_2_ influences the drug delivery process, the cell response to anatase or rutile was inconclusive [38,39,40]. Some studies confirmed the improvements in the adhesion and proliferation of cells regarding the annealed ATO samples compared with amorphous ones [39], while others demonstrated an adverse effect of fluoride ions regarding cell proliferation [38,40]. Nonetheless, the anatase phase’s enhanced impact on the cell response may be attributed to a better wettability of annealed TiO_2_ layers [40].

Considering the above, the impact of the annealing process on modifying ATO samples with NaOH was studied. Moreover, the effects of the further functionalization of such layers with different silane derivatives (i.e., (3-aminopropyl)triethoxysilane (APTES), (3-glycidyloxypropyl)trimethoxysilane (GPTMS), and (3-mercaptopropyl)trimethoxysilane (MPTMS)) on the ibuprofen delivery process and MG-63 cells response were examined. Complex research on the influences of functionalization of annealed TiO_2_ layers on the interactions with osteoblast-like cells and drug molecules has not yet been presented to the best of our knowledge.

## 2. Results and Discussion

### 2.1. The Effect of the Crystalline Structure on the ATO Modification with NaOH

**Figure 1:**
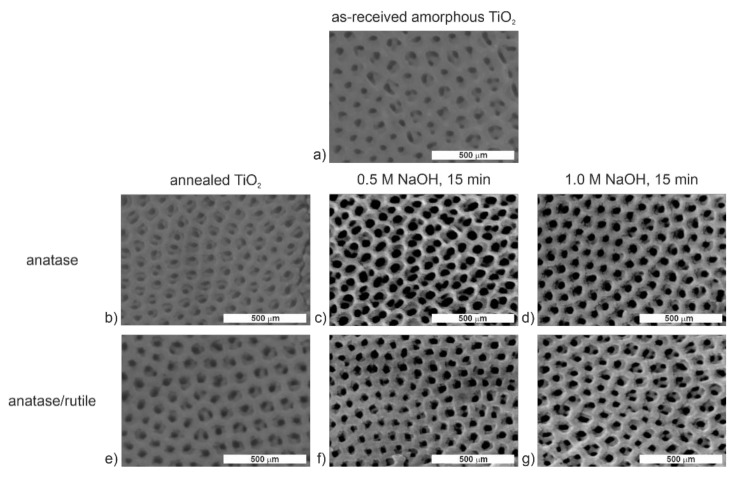
FE-SEM images of nanoporous TiO_2_ layers non-modified (**a**,**b**,**e**) and modified with 0.5 M (**c**,**f**) or 1.0 M (**d**,**g**) NaOH for 15 min. The TiO_2_ layers were non-annealed (**a**) and annealed at 400 (**b**–**d**) or 600 °C (**e**–**g**) for 2 h before the surface modification with NaOH.

The literature shows that annealed titania samples exhibit better bioactivity and interaction with osteoblastic cells [38,40,41]. While most articles have been focused mainly on the modification of amorphous ATO samples [9,10], we have decided to examine the influence of the annealing process on the effectiveness of the modification of titanium dioxide layers with NaOH. Based on our previous study [20], two annealing temperatures (400 and 600 °C) were selected for obtaining the anatase and mixed anatase and rutile phases without destruction of the nanoporous structure. For successful modification of TiO_2_ layers, two approaches were applied—i.e., (i) the samples were annealed prior the immersion in NaOH; (ii) the amorphous NaOH-modified samples were annealed at the temperatures of 400 or 600 °C. The SEM micrographs of the samples modified according to the procedure (i) are shown in Figure 1.

**Figure 2:**
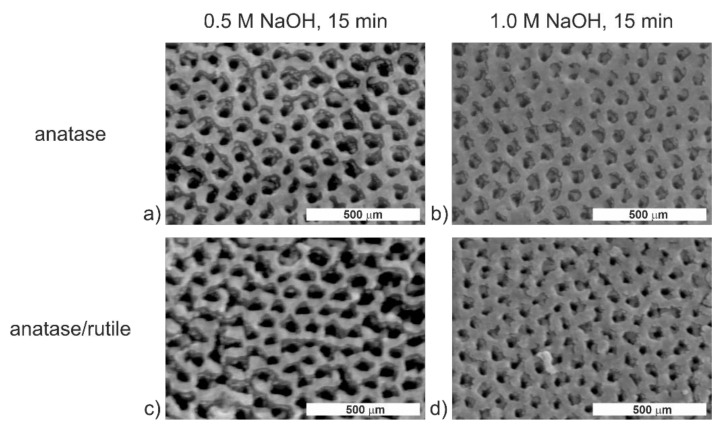
Microphotographs from the FE-SEM of ATO layers modified with 0.5 M (**a**,**c**) or 1.0 M (**b**,**d**) NaOH for 15 min. The TiO_2_ layers were annealed at 400 (**a**,**b**) or 600 °C (**c**,**d**) for 2 h after the modification with NaOH.

Based on FE-SEM images for the annealed TiO_2_ layers (Figure 1), it can be stated that, contrary to the amorphous anodic TiO_2_ [11], soaking in NaOH solution did not lead to any significant change in the surface morphology. Moreover, even when a more concentrated sodium hydroxide solution (Figure 1d,g) was used, the surface morphology did not change like it did for the amorphous layers [11]. Figure S1 and Table S1 show the chemical composition of studied TiO_2_ layers based on the EDS analyses. In general, the EDS spectra of ATO layers showed only Ti and O peaks, and the calculated amounts of sodium and fluorine were both equal to 0 within the margin of error. It indicates that the annealed TiO_2_ samples cannot be modified effectively with sodium hydroxide using the proposed experimental procedure, which is in contradiction to the research conducted by Morgado et al. [42], where titanate nanotubes were formed by hydrothermal treatment of an anatase powder with a concentrated NaOH solution (10 M) at moderate temperatures (90–170 °C). Therefore, it can be concluded that the surface modification of annealed TiO_2_ layers can be effective only at higher NaOH concentrations and temperatures, and extended modification times. However, as we have previously demonstrated, the NaOH concentration increase may result in the deterioration of TiO_2_ nanoporosity [11], which is a disadvantage. Therefore, the modification sequence was changed so that the annealing process was conducted after the modification with NaOH. FE-SEM images of the obtained nanostructures are presented in Figure 2.

FE-SEM micrographs for such modified TiO_2_ layers show slight differences in the surface morphology compared to the non-modified substrates (Figure 1a). Their surfaces look more roughened than as-prepared ATO layers, though the nanoporous structure of ATO is not destroyed under any of the applied conditions. The performed EDS analyses revealed that, apart from Ti and O peaks, the Na peak was present in the spectra (Figure S2). Besides, the Na content was at the same level for both the non-annealed and annealed samples [11]. As in the previous case, fluorine was absent in the studied substrates (Table S2). The above results confirm that the proposed procedure of modification is effective. Considering the previous studies [25,26], which demonstrated improved drug release kinetics from the anatase samples, TiO_2_ layers modified with 0.5 M NaOH for 15 min and annealed at 400 °C for 2 h (called Nan) were used for further studies.

### 2.2. Surface Modifications of Annealed TiO_2_ Layers with Silane Derivatives

As mentioned, various functional groups on the surface affect ATO layers’ interactions with drug molecules and cells [16,17]. Therefore, the Nan samples were further modified with silane derivatives, namely, APTES, GPTMS, and MPTMS. Based on our previous study [11] on the modification of amorphous ATO layers with silane derivatives, we applied the same procedure for the annealed NaOH-modified ATO samples. Briefly, samples were immersed in a 1% APTES, GPTMS, or MPTMS solution for 2 h. Absolute ethanol was used as a solvent because even a small amount of water may convert silane derivatives into silanes. Moreover, computer simulations showed that the adsorption of water molecules on the TiO_2_ surface could be competitive with the adsorption of organic molecules [39].

**Figure 3:**
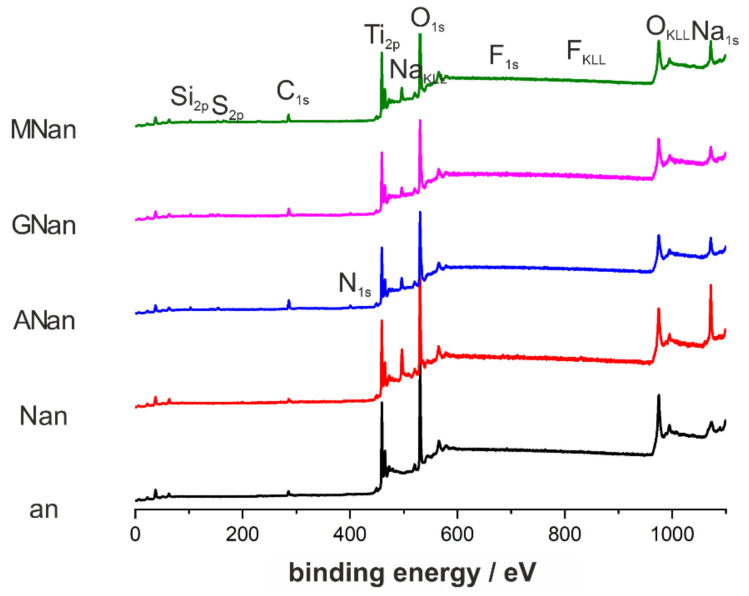
XPS (X-Ray Photoelectron Spectroscopy) survey spectra of the non-modified (an) and modified (Nan, Anan, GNan, and MNan) TiO_2_ samples.

The silane derivatives are small molecules, and the immersion time is short, so it can be expected that the samples would be coated with monolayers. Due to this fact, the elemental composition of ATO surfaces was characterized by using X-ray photoelectron spectroscopy, and the results are shown in Figure 3 and collected in Table 1.

Since XPS is a technique used to analyze the surface chemistry of materials, the differences in the atomic contents of characteristic elements for the tested samples were observed. As expected, the fluorine content was low for annealed samples because the heat treatment resulted in removing those anions from the ATO layers. For the non-modified and NaOH-modified ATO layers, carbon’s presence can be attributed to adventitious carbon contamination or carbon species embedded in the ATO structure, e.g., during the anodization process. In contrast, for the silane-modified samples, higher C content was strictly related to the modification process. The effectiveness of surface modification was confirmed by silicon peaks (Figure 3) in all XPS spectra for silane derivative-modified samples. It is worth noting that the sodium content decreased considerably after the modification with silane derivatives (Table 1), which confirms that the surface of titanium dioxide was covered with other molecules. Additionally, for more in-depth chemical analysis of the modified TiO_2_ layers, the detailed XPS spectra for nitrogen, sulfur, silicon, oxygen, and carbon are presented in Figures S3–S7, Supplementary Information. Additionally, explanations of the particular signals in each core-level XPS spectra are given there, confirming the successful modification of ATO substrates with silane derivatives.

One of the factors that significantly affect cell adhesion is the wettability of the biomaterials [30,35,43] due to the adhesion of water and proteins [4,12]. Their adsorption occurs immediately after the implant is placed in the patient’s body, so the biomaterial surface should be hydrophilic [4,12,35,43]. For this reason, the wettability of modified ATO samples was determined. The average values of the contact angle with the standard deviation of the mean for *n* = 20 are presented in Table 2.

As shown in Table 2, contact angle values were significantly less than 90°, proving the hydrophilic character of the samples. All the annealed substrates were more hydrophilic than as-prepared amorphous TiO_2_ layers [11], which is consistent with the literature data [4,44]. Such behavior may be explained in terms of the increased nano- and micro-roughness of the annealed surface and the presence of the anatase phase itself [44]. It should also be pointed out that the hydrophilicity of nanostructured TiO_2_ also depends on time (the so-called aging process) [44], storage conditions (e.g., access to air and temperature) [45], and anodization conditions (e.g., applied voltage, electrolyte composition, and substrate used) [46]. In the case of the samples used in the present work, only freshly prepared samples were applied for all the experiments. However, from our laboratory experience, we observed that annealed and aged samples (even after one year of storage at room temperature) remained hydrophilic (contact angles were in the range of 40–50°). This is also consistent with the work of Shin et al. [44], who showed that for annealed titania nanotubes formed on the pure Ti foil, the contact angle increased by 15° after three months of the storage.

Further surface modifications led to the enhancement of hydrophilic properties. Among them, NaOH-modified layers were the most hydrophilic (14.3 ± 2.2). The different wettability levels of the examined samples were caused by their chemical surface properties caused by various functional groups. The improvement of the TiO_2_ surface’s wettability after their immersion in NaOH may be correlated with the presence of hydroxyl groups. The functionalization with silane derivatives slightly increases the contact angle, when compared with NaOH-modified samples (29.1 ± 3.0, 20.3 ± 2.7, and 18.6 ± 3.9, for MPTMS, APTES, and GPTMS, respectively). It is consistent with the observations from Lin et al. [17], suggesting that the ATO layers with thiol groups at the surface were more hydrophobic than those with amino groups. Based on the above results, it can be concluded that all examined samples may exhibit good adsorption of proteins from the medium, and consequently, sufficient cell adhesion can be expected.

### 2.3. Effects of the Surface Modification on Ibuprofen Release Kinetics

As presented in our previous studies [11,25,26], drugs with different structures and water-solubility can be released from TiO_2_ nanopores. Such a drug delivery process is described with the desorption-desorption-diffusion (DDD) kinetic model proposed and described by Jarosz et al. [25]. The DDD model involves the desorption of the drug molecules from the TiO_2_ surface, followed by the desorption and diffusion of the molecules from the nanoporous structure [25]. This model may be applied for both single [25] and double-drug release kinetics [26], as well as for the release process from ATO layers with different crystalline structures [25,26] and surface modification [11].

**Figure 4:**
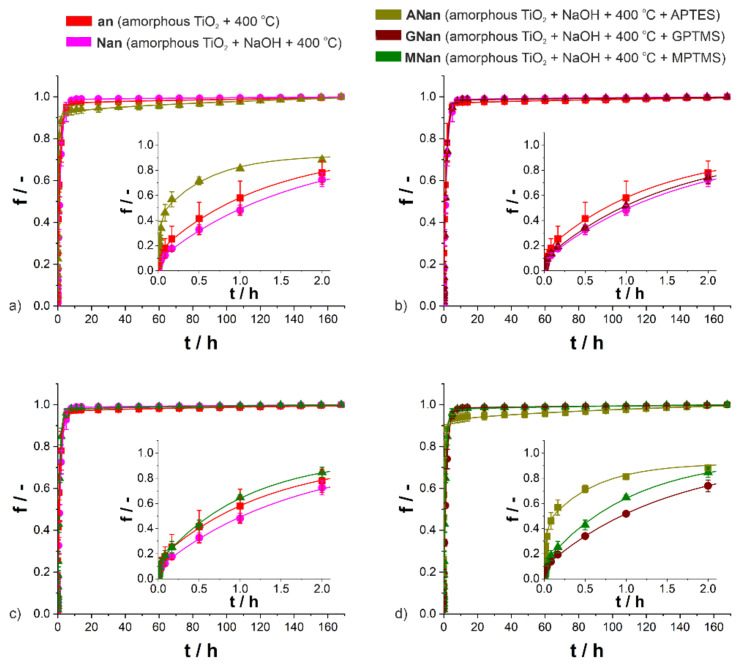
Drug release profiles of ibuprofen from the non-modified (an) and modified (Nan, Anan, GNan, and MNan) TiO_2_ samples with the fitted DDD model ((**a**) an, Nan, and Anan; (**b**) an, Nan, and GNan; (**c**) an, Nan, and MNan; (**d**) ANan, GNan, and MNan). Insets show the first 2 h of the release process (the standard deviations of the means, represented as bars, were calculated for *n* = 3).

Figure 4 presents the ibuprofen release profiles according to the DDD kinetic model. The characteristic parameters describing the process are collected in Table S3. Additionally, the amounts of ibuprofen released at predetermined time points from the samples are shown in Table S4, and the cumulative masses of the drug released after 168 h from the samples are reported in Table S5.

As expected, biphasic release behavior was observed for all types of systems (Figure 4). An initial burst release stage, which occurred immediately after the immersion in the PBS, was followed by slow ibuprofen delivery [25]. As shown in Table S3, the correlation coefficients were equal to 0.999, which proved the accuracy of the DDD model fitting. In contrast to amorphous and functionalized ATO samples [11], no apparent effect of the surface functionalization on the ibuprofen release process and its kinetic parameters could be seen (Figure 4 and Table S3). As proved before [25], the presence of the anatase phase solely caused the significant reduction in the rate of ibuprofen delivery process, especially the decrease in the amount of drug released during the first stage. When modified with silane derivatives, only in the case of APTES were differences in the release kinetics observed, which may be correlated with a significantly lower amount of loaded ibuprofen for those samples (0.9 mg) than for others (1.9–3.0 mg) (Table S5). Based on the detailed analysis of kinetic parameters (Table S3), it can be stated that apart from the ANan samples, the parameters f_1_ and k_1_, which describe the first stage of the ibuprofen release process, take lower values for the modified and annealed TiO_2_ layers. For all samples, the parameter f_2_ was above 0.910, so it can be assumed that the drug release process was completed. The parameter k_2_ (i.e., the first-order kinetic constant for the second stage of the process) is ascribed to the kinetics of desorption from the inner surface of nanopores. The values for NaOH-modified and GPTMS-modified samples were higher, while the values for APTES and MPTMS-modified samples were lower than those for only annealed titania. The reason for such behavior was the presence of -NH_2_ and -SH groups, which may slightly inhibit the process. Moreover, when the dissolution constant (K_H_) is considered, only modification with APTES can result in an increase in the diffusion of drug molecules inside nanopores. As shown in Table S5, for all five types of studied DDSs, the amount of drug released after a week is similar within the margin of error. Considering the above, although some differences may be observed, the functionalizations of annealed TiO_2_ layers with NaOH and silane derivatives (APTES, GPTMS, and MPTMS) have little or no effect on the ibuprofen delivery.

### 2.4. Biocompatibility of the Modified ATO Layers

**Figure 5:**
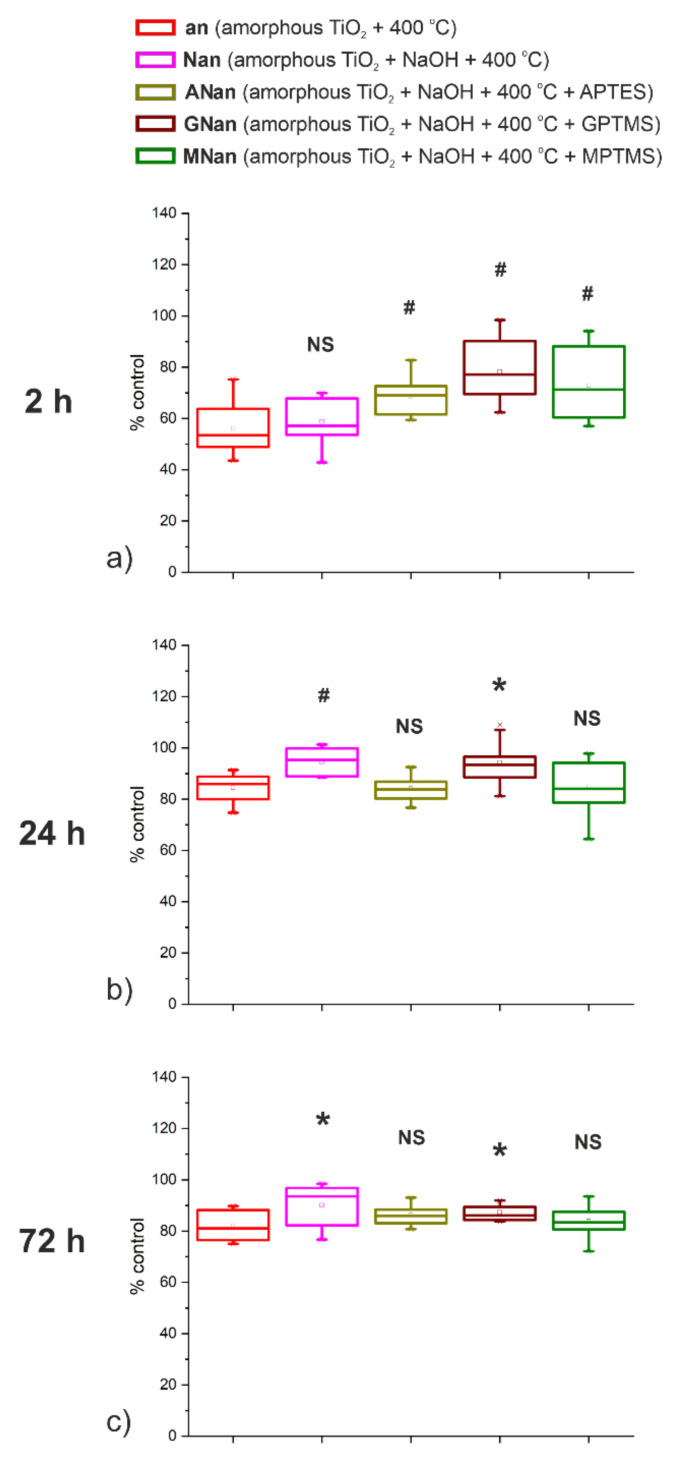
Metabolic activity of osteoblast-like cell line MG-63 incubated on the non-modified (an) and modified (Nan, Anan, GNan, and MNan) TiO_2_ samples after 2 (**a**), 24 (**b**), and 72 (**c**) hours. The values are expressed as percentages of the positive control (PS); NS—not statistically significant differences, *—statistically significant differences at the alpha level of 0.05, #—statistically significant differences at the alpha level of 0.01, based on ANOVA.

We recently showed that the modification of amorphous TiO_2_ layers with NaOH and APTES improves the interaction between the samples and osteoblast-like cells line SAOS-2 [11]. In new studies, a cell line with a different phenotype (MG-63) has been chosen [47]. The MTS test, a one-step colorimetric method, was used to measure the cell metabolic activity after 2, 24, and 72 h incubations of MG-63 cells on the selected samples. The results are presented in Figure 5.

The MG-63 cells were incubated on the studied samples to analyze their adhesion, growth, and proliferation (Figure 5). After 2 h of cultivation, for all surfaces, the cells’ metabolic activity was in the range of 40–80% of the activity of control cells cultivated on PS (polystyrene) (Figure 5a), which means that the initial cell adhesion to the modified surfaces was less effective. While the presence of -OH groups at the surface has no impact on the activity of the cells, significant differences in their metabolism were observed for the anodic TiO_2_ layers modified with silane derivatives (Figure 5a). At this point, only adhesion of cells took place, so its slower rate may have caused the observed differences. After the 24 h incubation, the cell’s activity reached the PS level, though the statistical significance was observed only for NaOH and GPTMS modifications (Figure 5b). The same tendency was noticed for the 72 h cultivation (Figure 5c). Such behavior may be explained with regard to the wettability of the samples shown earlier, where the highest hydrophilicity was observed for those two types of modifications. It proves that wettability plays a crucial role in the adsorption of the molecules and in cell adhesion. Considering the above, it may be concluded that surface modification with GPTMS affects the adhesion and proliferation of MG-63 cells the most. Similar improvement in biocompatibility for such a change has also been observed for the L-929 fibroblasts cell line [48]. Therefore, it can be assumed that GPTMS-modified ATO layers will be promising in terms of using titanium-based implants.

**Figure 6:**
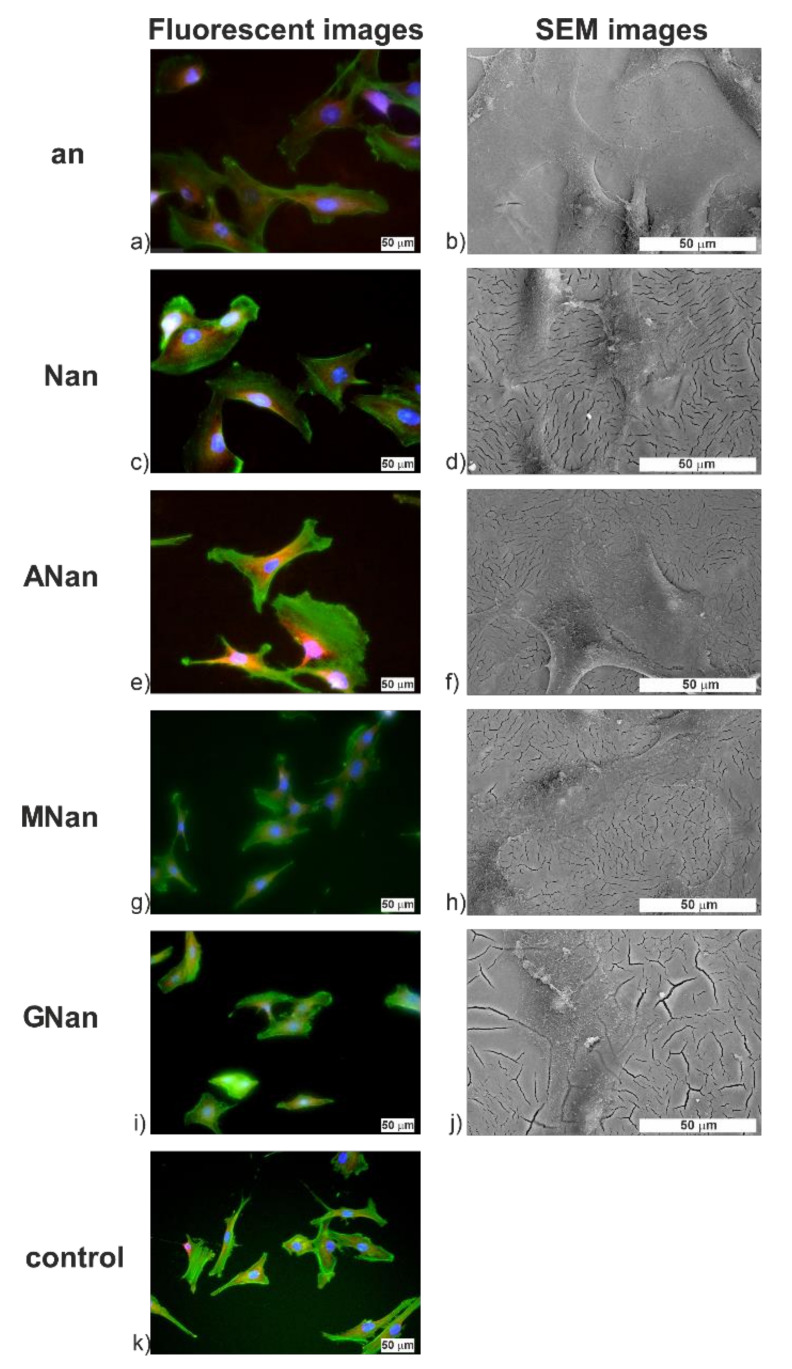
Representative immunofluorescence and FE-SEM microphotographs of MG-63 osteoblast-like cells grown on the non-modified (an) (**a**,**b**) and modified (Nan, Anan, GNan, and MNan) (**c**–**j**) TiO_2_ samples, and the control (polystyrene) (**k**) after 24 h of culture. For fluorescent microscopy imaging, cells were stained with actin skeleton (green), cell nuclei (blue), and vinculin (red). Scale bar = 50 μm.

The immunofluorescence images and FE-SEM images of MG-63 cells after 24 h of incubation showing the morphology of adhered cells are presented in Figure 6; those after 2 and 72 h are shown in Figure S8. After 24 h (Figure 6), the cells were well spread on all samples with clearly marked actin filaments. This is also supported by the FE-SEM images, where well-flattened cells with clearly visible filopodia may be seen (Figure 6b,d,f,h,j), which indicates good cell adhesion due to the presence of appropriate adhesive sites [49]. The same observations were reported by Lin et al. [17] for 3T3 fibroblasts on anodic non-modified and silane derivative-modified TiO_2_ layers. No significant differences in the morphology of the MG-63 cells were observed, proving the biocompatibility of the proposed modified ATO layers.

To sum up, it may be stated that all of the proposed samples are biocompatible and facilitate the adhesion and proliferation of osteoblast-like cells. However, the most significant changes after 24 and 72 h of incubation may be observed only for the samples modified with NaOH and GPTMS, proving that surface groups (i.e., -OH and glycidyl) play essential roles in the interactions between the material and cells.

## 3. Materials and Methods

### 3.1. Synthesis of Nanostructured Titanium Dioxide on Ti Substrate

Rectangular Ti samples with dimensions of 1 cm × 2 cm were first degreased in acetone and ethanol, and then polished electrochemically (in the mixture of acetic, sulfuric, and hydrofluoric acids) and chemically (in the mixture of hydrofluoric and nitric acids) [50]. After being rinsed with distilled water and ethanol, the Ti samples were dried in the air. Nanoporous TiO_2_ layers were prepared via a three-step anodization process in a two-electrode cell with titanium foils as both the anode and cathode. Details regarding the standard anodization parameters applied in this work may be found in our previous papers [51,52,53]. As a result, the TiO_2_ films with a suitable pore arrangement were synthesized.

### 3.2. Modification of Surface and Crystalline Structure of TiO_2_ Layers

Two approaches were used to examine how the crystalline structure of TiO_2_ affects the surface modification of ATO layers with sodium hydroxide. Firstly, the samples were annealed at the temperature of 400 or 600 °C for 2 h in a muffle furnace (model FCF5-SHM Z, Czylok, Jastrzębie-Zdrój, Poland) to obtain the anatase and a mixture of anatase and rutile phases, respectively. After the heat treatment, the TiO_2_ layers were immersed in NaOH (0.5 or 1.0 M) for 15 min and rinsed with distilled water. In the second approach, TiO_2_ layers were first immersed in sodium hydroxide and then annealed. The morphology of samples was examined by field emission scanning electron microscopy with energy-dispersive X-ray spectroscopy (FE-SEM/EDS, Hitachi S-4700 with a Noran System 7, Krefeld, Germany).

Subsequent surface changes with silane derivatives (APTES, GPTMS, and MPTMS) were achieved by immersing the NaOH-modified and annealed samples in a 1% ethanolic solution silane derivatives for 2 h and rinsing with absolute ethanol. The characterization of the modified nanostructured TiO_2_ was performed using X-ray photoelectron spectroscopy (ESCA/XPS) with an Al X-ray source (1486.7 eV) and a semi-spherical analyzer SES R4000 (Gammadata Scienta, Uppsala, Sweden). The maximum energy resolution for the Ag 3d_5/2_ line was 1.0 eV (for analyzer pass energy of 100 eV). The curves were fitted with a Voigt profile (GL = 30) and a Shirley background using CasaXPS software 2.3.15.

The contact angle was measured using a sessile drop technique (goniometer DataPhysics OCA25 equipped with a CCD device and computer, Filderstadt, Germany) to determine the effect of the surface modification on the wettability of TiO_2_ layers. The contact angle was measured 10 times for each deionized water droplet. The procedure was repeated three times for each type of sample.

### 3.3. Ibuprofen Delivery Process

An ibuprofen release process from anodic TiO_2_ layers was examined for selected samples. A standardized loading strategy described previously was applied [25,26]. Briefly, 1 mL of a 10 wt.% solution of ibuprofen in ethanol (Polfarmex-Kutno, Kutno, Poland) was pipetted fivefold onto the anodic TiO_2_ layers, followed by air drying at room temperature. Afterward, the ibuprofen excess was removed from the surface with the paper towel, and samples were weighed after a 24 h-storage in a desiccator.

In vitro drug delivery was carried out for a week according to the optimized procedure [25,26]. The drug-loaded sample was immersed in the 0.01 M phosphate buffer solution (PBS, pH = 7.4) at a temperature of 37 ± 1 °C. The whole volume of PBS (6 mL) was replaced with a fresh portion of the solution at predetermined time points. The ibuprofen concentration was determined based on the UV-Vis spectra recorded at the wavelength range of 190–300 nm (Thermo Scientific Evolution 220 coupled with Thermo Scientific Insight software, Waltham, MA, USA) and then calculated based on the absorbance measurements at 222 nm and constructed calibration curve. The desorption-desorption-diffusion (DDD) model was fitted to the obtained data to determine the kinetics of the process.

### 3.4. Cell Cultivation on Studied ATO Layers

The human osteoblast-like cell line MG-63 (ATCC®CRL-1427, LGS Standards, UK) was cultivated in DMEM (Dulbecco’s modified eagle’s medium) with glucose, phenol red, and L-glutamine (Sigma-Aldrich, St. Louis, MI, USA) supplemented with 10% heat-inactivated fetal bovine serum (FBS) (HyClone, Logan, UT, USA), penicillin (10 U mL^−1^, HyClone), and streptomycin (10 μg mL^−1^, HyClone)) at 37 °C in a 5% CO_2_ atmosphere. The biological experiments were performed for five types of samples: an, Nan, Anan, GNan, and MNan. Firstly, the samples were exposed to UV light for 15 min in order to sterilize them. The cells were seeded on ATO layers in 24-well tissue culture plates, and tissue culture polystyrene (PS) was used as a control. In the biological studies, a seeding density of 10,000 cells cm^−2^ was used. The seeded MG-63 cells were incubated for 2, 24, and 72 h.

### 3.5. Examination of the Metabolic Activity and Morphology of MG-63 Cells

The CellTiter 96^®^ Aqueous One Solution Cell Proliferation Assay (MTS, Promega, Madison, WI, USA) was applied to determine the number of viable cells. After 2, 24, or 72 h, the medium was removed, and a 10% MTS solution in the DMEM medium was added. After the next 2-h incubation at 37 °C, 100 μL of 10% MTS solution in DMEM was transferred into a 96-well plate (in triplicates for each sample). In the end, the absorbance at 490 nm was measured with a multi-detection micro-plate reader (Epoch™ 2, BioTek, Winooski, VT, USA). The results were expressed as ratios of absorbance measured for the TiO_2_ samples to absorbance of the control (tissue culture PS). The statistical analysis was based on twelve results for each type of sample obtained from two independent experiments. The evaluation was carried out using one-way analysis of variance (ANOVA) (significance assessed at *p* < 0.05) in R Studio software [54].

The cells were immunofluorescently labeled after the same incubation times to determine their morphology on the substrates, according to the previously shown procedure [11]. The cells were fixed in 4% paraformaldehyde in PBS for 15 min and then rinsed with PBS. To increase the cell membranes’ permeability, 0.1% Triton X-100 (Sigma-Aldrich, Poland) in PBS was added to each well and incubated for 20 min. After that, the cells were rinsed with PBS, and the blocking solution containing 1% FBS and 0.05% Tween^®^20 (Sigma-Aldrich, Poland) in PBS was added to block the non-specific binding sites. After a 30-min incubation, the solution was replaced with a primary antibody (mouse anti-vinculin monoclonal antibody) diluted in the blocking solution (1:500, Sigma-Aldrich), and incubated for 1 h at 37 °C. Next, the samples were rinsed with PBS. Afterward, secondary antibody, goat anti-mouse (1:500, Invitrogen, Carlsbad, CA, USA), and phalloidin-Alexa 488 (1:500, Invitrogen) in a blocking solution, were added and incubated at 37 °C for 45 min. After the rinse with PBS, the cell nuclei were labeled with 4′,6-diamidino-2-phenylindole dihydrochloride in PBS (DAPI, 1:1000, Sigma-Aldrich, Poland) and incubated for 15 min. In the end, the ATO layers were rinsed with PBS and mounted with a mounting medium (Thermo Scientific™ Shandon™ Immu-Mount™, Thermo Scientific). Fluorescence images of the stained MG-63 cells were obtained with the Olympus IX51 (Olympus, Warsaw, Poland) equipped with an XC10 camera.

Additionally, the morphology of the studied samples was examined by using the scanning electron microscope. ATO layers were firstly fixed with a 3% glutaraldehyde solution for 24 h. The samples were then rinsed three times with PBS and dehydrated in the alcohol series (the ethanol solutions with a concentration of 50%, 60%, 70%, 80%, 90%, 96%, and 100%). Non-modified and modified TiO_2_ were placed in each solution for 15 min. Finally, the substrates were dried for 1 min in the solution of hexamethyldisilazane [55]. Before the samples were characterized with SEM, they were coated with a 15-nm layer of gold using a sputter coater (Quorum Q150T S, UK).

## 4. Conclusions

This report has shown how structural and surface modifications of nanostructured TiO_2_ influence the biocompatibility and drug release properties.

It was found that anatase and mixed anatase and rutile phases could not be effectively modified with NaOH, whereas a crystalline structure change is possible for the NaOH-modified samples. Therefore, for effective and nondestructive modification, the sequence of the modification steps is crucial. Moreover, such samples may be further functionalized with silane derivatives having different functional groups. Both the heat treatment and surface functionalization improved the hydrophilicity of the studied ATO surfaces, which are crucial for biomedical applications.

Modified ATO layers were used to test the ibuprofen release from the nanopores. Unfortunately, the additional modifiers did not affect the release kinetics in any way. The most significant improvement was observed only for the annealed layers. On the other hand, the proposed modifications improved the cell responses on the studied substrates. The biological studies showed an increase in the metabolic activity of MG-63 cells and improvement in the cells’ morphology, especially when samples were modified with APTES and GPTMS.

To summarize, the presented data provide more information about the surface modification of nanostructured anatase layers. It can also be postulated that the modifications with APTES and GPTMS were the most beneficial for the potential implantable materials, which improve cell response and provide drug molecules directly at the implantation site.

## Figures and Tables

**Table 1:** Elemental compositions of non-modified (an) and modified (Nan, Anan, GNan, and MNan) TiO_2_ samples based on the XPS analyses.

Sample	Atomic Content [%]							
	C	O	Ti	F	Na	Si	N	S
an	11.14	63.70	24.61	0.55	-	-	-	-
Nan	9.12	59.28	19.05	0.72	11.84	-	-	-
ANan	19.72	50.75	15.85	0.00	4.32	6.29	3.07	-
GNan	19.03	54.49	16.94	0.18	3.89	5.47	-	-
MNan	14.57	55.07	17.68	0.44	4.62	4.08	-	3.53

**Table 2:** Contact angle measurements of the non-modified (an) and modified (Nan, Anan, GNan, and MNan) TiO_2_ samples.

Sample	Contact Angle [°]
an	41.1 ± 3.3
Nan	14.3 ± 2.2
ANan	20.3 ± 2.7
GNan	18.6 ± 3.9
MNan	29.1 ± 3.0

## Data Availability

Data sharing not applicable.

## Supplementary Materials

Figure S1: EDS spectra of nanoporous TiO2 layers annealed at 400 °C for 2 h and then immersed in 0.5 M (a) or 1.0 M (b) NaOH for 15 min. The Au peak in the spectra has arisen from a gold layer sputtered before SEM examination. Table S1: Elemental compositions (Ti, O, Na, and F) of TiO2 samples annealed at 400 °C for 2 h and then immersed in 0.5 or 1.0 M NaOH for 15 min, calculated based on EDS spectra. Figure S2: EDS spectra of nanoporous TiO2 layers modified with 0.5 M (a) or 1.0 M (b) NaOH for 15 min and then annealed at 400 °C for 2 h. The Au peak in the spectra has arisen from a gold layer sputtered before SEM examination. Table S2: Elemental composition (Ti, O, Na, and F) of TiO2 samples modified with 0.5 M or 1.0 M NaOH for 15 min and then annealed at 400 °C for 2 h, calculated based on EDS spectra. Figure S3: Core-level XPS spectra of N 1s for the ANan sample. Figure S4: Core-level XPS spectra of S 2p for the MNan sample. Figure S5: Core-level XPS spectra of Si 2p for the ANan (a), GNan (b), and MNan (c) samples. Figure S6: Core-level XPS spectra of O 1s for the an (a), Nan (b), ANan (c), GNan (d), and MNan (e) samples. Figure S7: Core-level XPS spectra of C 1s for the an (a), Nan (b), ANan (c), GNan (d), and MNan (e) samples. Table S3: The desorption-desorption-diffusion model parameters (f_1_, f_2_, k_1_, k_2_, K_H_) with parameter standard errors for the ibuprofen release from the modified and non-modified annealed TiO2 layers. Table S4: The amounts of ibuprofen released after pre-determined time points, namely, 10 s, 60 s, 10 min, 30 min, 1 h, 2 h, 24 h, and 168 h, from the modified and non-modified annealed TiO2 layers. Table S5: The amounts of ibuprofen loaded inside nanoporous TiO2 layers and released after 168 h from the modified and non-modified annealed TiO2 samples (with standard deviation, n = 3). Figure S8: Representative immunofluorescence images of MG-63 osteoblast-like cells grown on the an (anatase TiO2) (a,b), Nan (amorphous TiO2 + NaOH + 400 °C) (c,d), ANan (amorphous TiO2 + NaOH + 400 °C + APTES) (e,f), GNan (amorphous TiO2 + NaOH + 400 °C + GPTMS) (g,h), MNan (amorphous TiO2 + NaOH + 400 °C + MPTMS) (i,j), and control (polystyrene) (k,l) samples after 2 and 72 h of culture. Cells were stained with actin skeleton (green), cell nuclei (blue), and vinculin (red). Scale bar = 50 μm.

## Sample Availability

Not available.
