# Supplementary material for: The Impacts of Crystalline Structure and Different Surface Functional Groups on Drug Release and the Osseointegration Process of Nanostructured TiO2
Source: Molecules. 2021 Mar 19;26(6):1723. doi: 10.3390/molecules26061723 (PMC8003584; doi:10.3390/molecules26061723)
Supplement: Supplementary file 1 [file molecules-26-01723-s001.zip › molecules-1126886-supplementary.pdf]

molecules

MDPI

Supplementary Material

The Impacts of Crystalline Structure and Different Surface Functional Groups on Drug Release and the Osseointegration Process of Nanostructured TiO_2

Anna Pawlik [1], Magdalena Jarosz [1,*], Robert P. Socha [2] and Grzegorz D. Sulka [1]

[1] Faculty of Chemistry, Jagiellonian University, Faculty of Chemistry, Gronostajowa 2, 30387 Krakow, Poland; pawlika@chemia.uj.edu.pl (A.P.); sulka@chemia.uj.edu.pl (G.D.S.)
[2] Jerzy Haber Institute of Catalysis and Surface Chemistry, Polish Academy of Science, Niezapominajek 8, 30239 Krakow, Poland; ncsocha@cyf-kr.edu.pl
* Correspondence: jarosz@chemia.uj.edu.pl; Tel.: +48-126-862-520

1. EDS Analysis

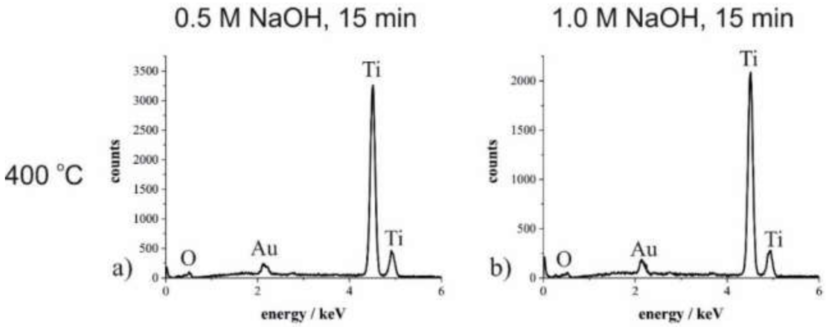

Figure S1. EDS spectra of nanoporous TiO_2 layers annealed at 400 °C for 2 h and then immersed in 0.5 M (**a**) and 1.0 M (**b**) NaOH for 15 min. The Au peak in the spectra has arisen from a gold layer sputtered before SEM examination.

Table S1. Elemental composition (Ti, O, Na, and F) of TiO_2 samples annealed at 400 °C for 2 h and then immersed in 0.5 M and 1.0 M NaOH for 15 min, calculated based on EDS spectra.

NaOH Concentration [mol·dm⁻³]	Annealing Temperature [°C]	Content [wt%]				Content [at%]			
		Ti	O	Na	F	Ti	O	Na	F
0.5	400	76.4 ± 0.5	16.2 ± 0.7	0.1 ± 0.2	0.3 ± 0.4	60.2 ± 0.4	38.2 ± 1.8	0.1 ± 0.3	0.6 ± 0.8
	600	77.9 ± 0.6	14.3 ± 1.0	0.0 ± 0.0	0.5 ± 0.5	63.6 ± 0.5	34.9 ± 2.3	0.0 ± 0.0	1.0 ± 1.0
1.0	400	79.3 ± 0.6	15.1 ± 1.2	0.1 ± 0.1	0.1 ± 0.4	62.9 ± 0.5	35.9 ± 2.9	0.2 ± 0.2	0.3 ± 0.4
	600	71.4 ± 0.6	18.9 ± 1.3	0.0 ± 0.0	0.0 ± 0.1	54.8 ± 0.5	43.45 ± 3.0	0.0 ± 0.0	0.0 ± 0.1

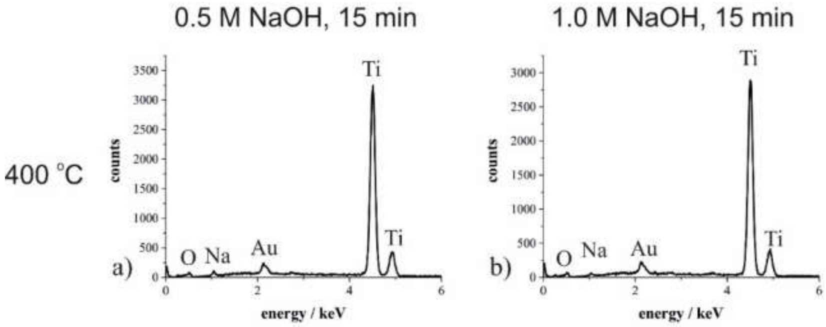

Figure S2. EDS spectra of nanoporous TiO_2 layers modified with 0.5 M (**a**) and 1.0 M (**b**) NaOH for 15 min and then annealed at 400 °C for 2 h. The Au peak in the spectra has arisen from a gold layer sputtered before SEM examination.

Table S2. Elemental composition (Ti, O, Na, and F) of TiO_2 samples modified with 0.5 M and 1.0 M NaOH for 15 min and then annealed at 400 °C for 2 h, calculated based on EDS spectra.

NaOH Concentration [mol·dm^{-3}]	Annealing Temperature [°C]	Content [wt%]				Content [at%]			
		Ti	O	Na	F	Ti	O	Na	F
0.5	400	77.0 ± 0.5	13.5 ± 0.7	2.2 ± 0.1	0.3 ± 0.3	62.3 ± 0.4	32.6 ± 1.8	3.7 ± 0.2	0.6 ± 0.8
	600	76.7 ± 0.6	13.1 ± 1.2	2.0 ± 0.1	0.0 ± 0.0	62.9 ± 0.5	32.1 ± 2.9	3.4 ± 0.2	0.0 ± 0.0
1.0	400	76.7 ± 0.6	15.0 ± 1.1	1.3 ± 0.2	0.1 ± 0.4	60.8 ± 0.4	35.1 ± 2.5	2.1 ± 0.3	0.2 ± 0.6
	600	73.2 ± 0.6	15.8 ± 1.1	3.2 ± 0.2	0.0 ± 0.0	56.7 ± 0.4	36.6 ± 2.6	5.2 ± 0.3	0.0 ± 0.0

2. XPS analysis

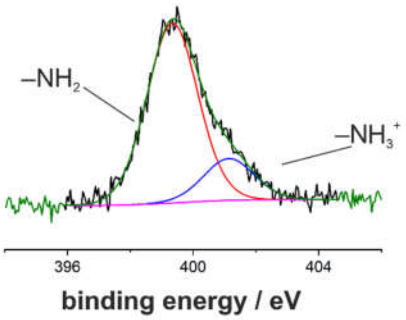

Figure S3. Core-level XPS spectra of N 1s for the ANan sample.

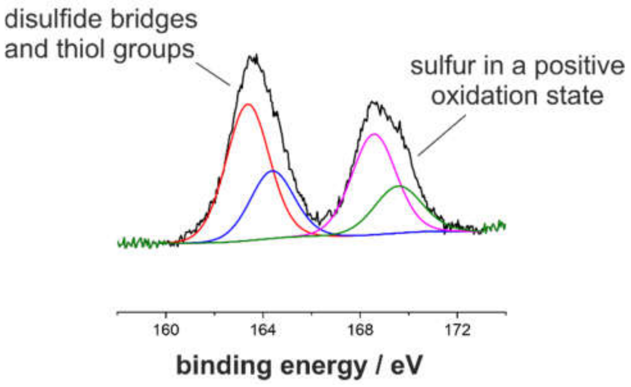

Figure S4. Core-level XPS spectra of S 2p for the MNan sample.

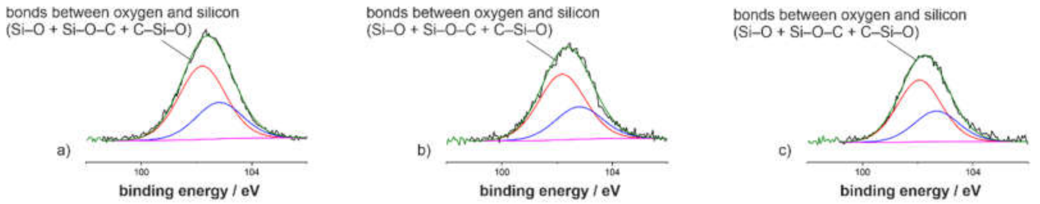

Figure S5. Core-level XPS spectra of Si 2p for the ANan (**a**), GNan (**b**) and MNan (**c**) samples.

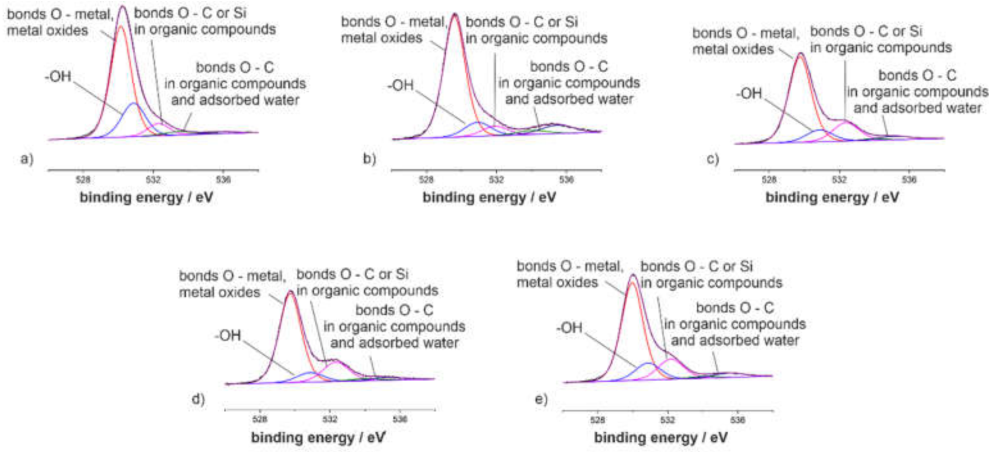

Figure S6. Core-level XPS spectra of O 1s for the an (**a**), Nan (**b**), ANan (**c**), GNan (**d**) and MNan (**e**) samples.

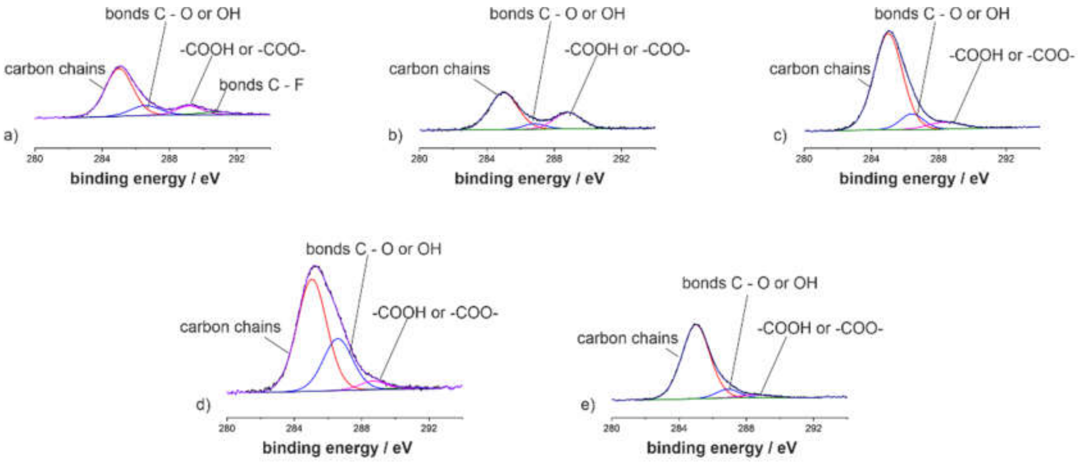

Figure S7. Core-level XPS spectra of C 1s for the an (**a**), Nan (**b**), ANan (**c**), GNan (**d**) and MNan (**e**) samples.

3. Ibuprofen Release Kinetics

Table S3. The desorption-desorption-diffusion model parameters (f_1, f_2, k_1, k_2, K_H) with parameter standard errors for the ibuprofen release from the modified and non-modified annealed TiO_2 layers.

Sample	f_1 [-]	f_2 [-]	k_1 [h^{-1}]	k_2 [h^{-1}]	K_H [$h^{-0.5}$]	R^2
an	0.150 ± 0.005	0.962 ± 0.003	35 ± 2	0.75 ± 0.01	0.0031 ± 0.0004	0.999
Nan	0.084 ± 0.004	0.983 ± 0.003	56 ± 6	0.60 ± 0.01	0.0014 ± 0.0004	0.999
ANan	0.418 ± 0.014	0.910 ± 0.006	49 ± 38	1.70 ± 0.11	0.0133 ± 0.0016	0.999
GNan	0.092 ± 0.002	0.980 ± 0.002	75 ± 4	0.65 ± 0.01	0.0015 ± 0.0002	0.999
MNan	0.123 ± 0.004	0.973 ± 0.003	56 ± 4	0.93 ± 0.01	0.0024 ± 0.0003	0.999

Table S4. The amount of ibuprofen released after pre-determined time points, namely 10, 60 s, 10, 30 min, 1, 2, 24, and 168 h, from the modified and non-modified annealed TiO_2 layers.

Sample	Mass of Released Ibuprofen [mg]							
	**10 s**	**60 s**	**10 min**	**30 min**	**1 h**	**2 h**	**24 h**	**168 h**
an	0.03 ± 0.01	0.15 ± 0.06	0.47 ± 0.14	0.8 ± 0.2	1.1 ± 0.2	1.5 ± 0.1	1.9 ± 0.2	1.9 ± 0.2
Nan	0.04 ± 0.02	0.18 ± 0.05	0.53 ± 0.08	1.0 ± 0.1	1.4 ± 0.2	2.2 ± 0.2	3.0 ± 0.6	3.0 ± 0.6
ANan	0.04 ± 0.01	0.22 ± 0.02	0.48 ± 0.07	0.6 ± 0.1	0.7 ± 0.2	0.8 ± 0.2	0.8 ± 0.2	0.9 ± 0.2
GNan	0.06 ± 0.01	0.20 ± 0.04	0.51 ± 0.05	0.9 ± 0.1	1.4 ± 0.2	2.0 ± 0.4	2.7 ± 0.4	2.7 ± 0.4
MNan	0.04 ± 0.01	0.21 ± 0.04	0.57 ± 0.09	1.0 ± 0.2	1.6 ± 0.5	2.0 ± 0.6	2.4 ± 0.7	2.4 ± 0.7

As can be seen from Table S4, the mass of ibuprofen released during the first 10 min is similar for all types of studied drug delivery systems (0.47 mg–0.57 mg). Within the first hour, ~81% of ibuprofen was delivered from the ANan sample, while ~57%, ~47%, ~52%, and ~65% of the drug was transferred from the an, Nan, GNan, and MNan samples, respectively (Figure 4, insets).

Based on Table S3, it can be stated that apart from the ANan sample, the drug fraction released in the whole process (f_2) is close to 1 (0.910–0.983). The first-order kinetic constant for the 2nd stage (k_2) is lower for the ATO samples modified with only NaOH or with NaOH and GPTMS than for the non-modified drug delivery system. The proposed ways of surface functionalization, apart from the modification with APTES, lead to an increase in the value of the Higuchi dissolution constant.

Table S5. The amount of ibuprofen loaded inside nanoporous TiO_2 layers and released after 168 h from the modified and non-modified annealed TiO_2 samples (with standard deviation, $n = 3$).

Sample	Mass of Loaded Ibuprofen [mg]	Mass of Released Ibuprofen [mg]
an	1.9 ± 0.4	1.9 ± 0.2
Nan	2.9 ± 0.4	3.0 ± 0.6
ANan	0.9 ± 0.2	0.9 ± 0.2
GNan	2.4 ± 0.1	2.7 ± 0.4
MNan	2.1 ± 0.8	2.4 ± 0.7

4. MG-63 cells, Fluorescent Images

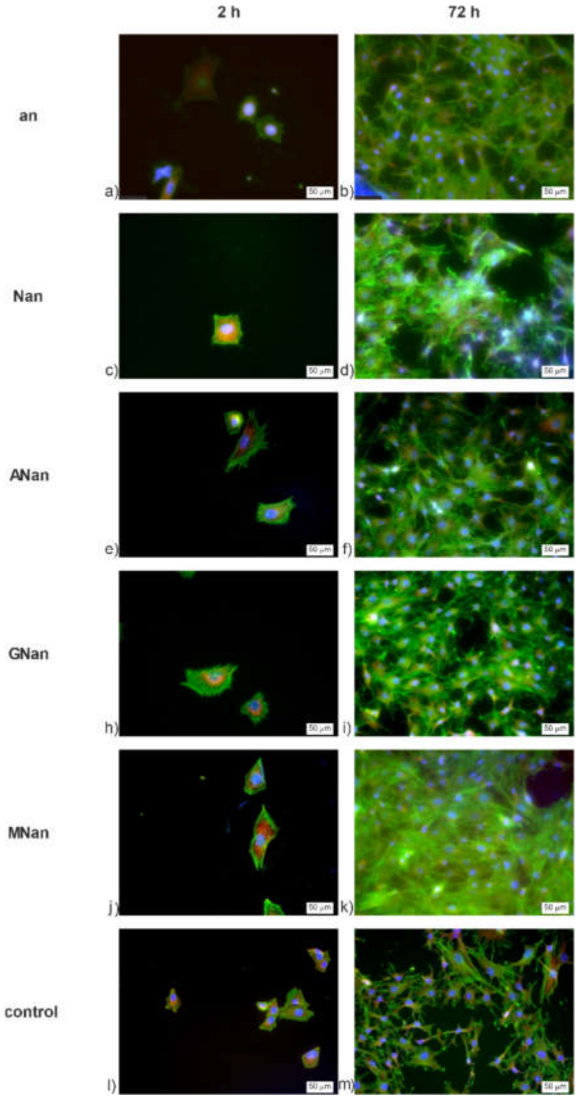

Figure S8. Representative immunofluorescence images of MG-63 osteoblast-like cells grown on the an (anatase TiO_2) (**a**,**b**), Nan (amorphous TiO_2 + NaOH + 400 °C) (**c**,**d**), ANan (amorphous TiO_2 + NaOH + 400 °C + APTES) (**e**,**f**), GNan (amorphous TiO_2 + NaOH + 400 °C + GPTMS) (g, h), MNan (amorphous TiO_2 + NaOH + 400 °C + MPTMS) (**i**,**j**) and control (polystyrene) (**k**,**l**) samples after 2 and 72 h of culture. Cells were stained with actin skeleton (green), cell nuclei (blue), and vinculin (red). Scale bar = 50 μm.
